# Enabling people with dementia to access and receive cancer treatment and care: The crucial role of supportive networks

**DOI:** 10.1016/j.jgo.2020.03.015

**Published:** 2020-09

**Authors:** Claire A. Surr, Rachael Kelley, Alys W. Griffiths, Laura Ashley, Fiona Cowdell, Ann Henry, Michelle Collinson, Ellen Mason, Amanda J. Farrin

**Affiliations:** a Centre for Dementia Research, School of Health and Community Studies, Leeds Beckett University, Leeds, UK; b School of Social Sciences, Leeds Beckett University, Leeds, UK; c Faculty of Health, Education and Life Sciences, Birmingham City University, Birmingham, UK; d Clinical Oncology, Leeds Teaching Hospitals NHS Trust, Leeds, UK; e Clinical Trials Research Unit, Institute of Clinical Trials Research, University of Leeds, Leeds, UK; f School of Medicine, University of Leeds, Leeds, UK

**Keywords:** Alzheimer's disease, Cancer, Dementia, Treatment, Care, Support, Ethnography, Family carers, Support networks, Oncology

## Abstract

**Objectives:**

Despite cancer and dementia being conditions in which prevalence increases with age, there remains limited research on the cancer treatment and care needs of this population. Our study aimed to address this gap and this paper reports on the role of supportive networks in enabling people with dementia to access cancer treatment and care.

**Materials and methods:**

An ethnographic study involving seventeen people with cancer and dementia, 22 relatives and nineteen oncology staff. It comprised observations (46 h) of and informal conversations during oncology appointments attended by people with dementia and their relatives and semi-structured interviews (*n* = 37) with people living with cancer and dementia, their relatives and staff working in various roles across oncology services. Data were analysed using thematic analysis.

**Results:**

Patients and oncology staff relied on and expected relatives to provide practical and emotional support around cancer treatment and care. Families varied in their ability to provide required support due to extent of the family network, practical issues, knowledge of the patient and their wishes, family conflict and the patient's willingness to accept help. Where no family network was available, support provision was complex and this could compromise access to cancer treatment.

**Conclusions:**

People with comorbid cancer and dementia rely heavily on a supportive family network to access treatment and care. Oncology services need to assess the supportive networks available to individual patients in developing cancer treatment plans. Urgent consideration needs to be given to how those with no family networks can be appropriately supported.

## Highlights

• Supportive networks are vital for people with dementia to access cancer treatment.
• Provision of such support by families is often expected by oncology staff.
• Some families have greater resources to provide support than others.
• Providing this support can lead to guilt, stress and worry.
• It is unclear who provides this support where there is no family network.

## 1 Introduction

Cancer and dementia can both lead to complex health and care needs and have increasing prevalence with age [1,2]. However, little research has focused on this dual-diagnosis population. International literature provides varying estimates of dementia prevalence in cancer populations [3]. A recent UK large dataset study concluded one in thirteen (7.5%) people aged 75+ with a cancer diagnosis also have a dementia diagnosis [4]. Thus a significant number of patients accessing cancer services have dementia. People with comorbid cancer and dementia (CCD) have complex needs, may experience worse outcomes, receive less treatment, and are more likely to experience complications from cancer treatment [5].

Due to the impact of dementia on a person's day-to-day living abilities, relatives often play an integral supportive role [6]. A review of interventions for older people receiving cancer treatment [7] identified few studies considering the support needs of relatives despite the known challenges and detrimental impact of caring for someone with cancer, dementia [8] or multimorbidity [9]. Families play an essential role in supporting their relative with dementia to negotiate hospital appointments and manage symptoms and side effects [6,10,11]. Little is understood about how people with dementia who have no or limited family support networks negotiate this. Cancer and dementia comorbidity studies have largely focused on family involvement in cancer treatment decision-making, with variable findings. Some relatives report having to assert their role to avoid being marginalised during decision-making, particularly when the person with CCD is unable to accurately provide this information [6]. However, this requires a delicate balance to ensure the perspective of the person with CCD is not then excluded [10], although research indicates people with CCD are often content to defer information-giving and decision-making to their relative [11]. A recent review concluded more research was needed on cancerdecision-making in CCD to improve support for clinicians and relatives [12].

This paper explores the role of supportive networks in assisting and enabling people with CCD to receive hospital-based cancer treatment and care. The data presented reflect a key theme that was developed within a larger study, whose aim was to understand the cancer treatment and care experiences and needs of people with CCD [13].

## 2 Materials and Methods

### 2.1 Methods

An ethnographic method was employed. Data were collected (by RK and AG) via observation within oncology services, semi-structured interviews, informal conversations and review of hospital medical notes. Individual, dyad or small-group (for family units of more than two members) interviews were conducted in private spaces, such as the family home or a hospital quiet room, based on participant preference. Interview topic guides, developed by the research team in collaboration with the study's Lay Advisory Group, asked about participants' experiences of cancer treatment and care for people with CCD. This group was comprised of four people affected by cancer and dementia; three were carers/former carers and one was a person living with both conditions. They were recruited via social media and the research team's existing networks. One was also a co-applicant on the grant proposal.

Non-participant observations of clinical areas of the hospital were conducted to develop familiarity with the setting and to understand care practices. Participant observations of appointments in oncology clinics (consultations, treatment, and follow up but not diagnostic appointments), alongside informal conversations took place with people with CCD and accompanying relatives. The researcher met the participants either at the hospital entrance or in the department waiting area and accompanied them throughout their time in the hospital, leaving them again at the hospital exit. Observations enabled an in-depth understanding of people's ‘in the moment’ experiences and supported inclusion of the direct experiences of individuals with moderate to severe dementia who could not take part in interviews. Informal conversations took place during the observations. They involved the researcher chatting with participant(s) about their experiences to better understand their perspective on what was being observed. Detailed field notes and pertinent information from medical records were recorded.

### 2.2 Sampling

The research was conducted in two English National Health Service (NHS) Trusts, consisting of three hospitals in two cities, which provide local cancer services (e.g. surgery, chemotherapy) (all sites) and more specialist regional provision (e.g. radiotherapy) (one site) and the surrounding community. Using purposeful sampling [14] we aimed to recruit people with CCD and relative participants with a range of cancer diagnoses, treatment experiences and demographics and staff members from a range of oncology roles. Where possible, this included key staff who had worked with those participating in observations. To provide a range of experiences throughout the cancer treatment and care journey, participants with CCD and relatives were also recruited through local support groups and via social media to gain the experiences of people who had completed cancer treatment within the last 5 years, and who may, offer additional perspectives, by reflecting back on their cancer care experiences. As this group would not have regular hospital appointments, they would have been challenging to recruit via NHS services.

### 2.3 Participants

Participants were people diagnosed with (or symptoms indicating suspected) dementia and cancer (of any type) who had or were undergoing cancer treatment (hospital recruited) or had completed this in the last five years (community recruited), their relatives (where available) or former carers (providing care in the last five years where the person had died), and staff members with recent/current experience of supporting people with dementia and cancer working within or supporting oncology services. Dementia diagnosis was determined by a recorded diagnosis in the patients records. Suspected dementia was determined by a score of 4+ on the Functional Assessment Staging Tool [15] as completed by a researcher in discussion with the potential participant/their family member. Participant Demographics are Summarised in Table 1.

### 2.4 Analysis

Data collection and analysis ran concurrently, informing the focus of subsequent data collection. It was conducted by members of the research team with input from two members of the Lay Advisory Group. We used ethnographically informed thematic analysis following an iterative process, which explored the content and patterns in the data via triangulation across all data sources [16]. Transcripts were read as a whole for a sample of interviews, before independent initial coding at a paragraph level (by RK, AG, FC and CS). Initial codes were grouped into broad areas to develop a coding framework, which was continually discussed and refined with additional lay members as further transcripts were analysed. The staff and person with dementia/relative interviews were initially analysed separately, before combining the coding trees to provide an overall thematic framework which was further refined and developed through coding the observational data and medical notes. On completion of coding, definitive themes were finalised through review and discussion.

**Table 1:** Participant demographics.

Characteristics	n (%)
**Participants with CCD (*n*** **= 17)**	
Female, n (%)	10 (59)
Cancer type, n (%)	
Lung	8 (47)
Prostate	4 (24)
Breast	1 (6)
Gastrointestinal	1 (6)
Other	3 (18)
Ethnicity	
White British	16 (94)
Hispanic	1 (6)
Age, mean (range)⁎ (*n* = 13)	75 (45–88)
Recruitment setting	
NHS	14 (82)

**Family caregivers (*n*** **=** **22)**	
Female, n (%)	14 (64)
Relationship to person with CCD	
Child	12 (55)
Spouse	7 (32)
Sibling	2 (9)
Grandchild	1 (5)
Recruitment setting	
NHS	19 (86)

**Staff (*n*** **=** **19)**	
Female, n (%)	14 (74)
Oncology role worked in	
Radiotherapy dept	7 (37)
Lung cancer clinic	6 (32)
Breast cancer clinic	3 (16)
Prostate cancer clinic	1 (5)
Other	2 (11)
Staff role	
Nurse	8 (42)
Radiographer	7 (37)
Consultant	2 (11)
Social worker	1 (5)
Patient transport officer	1 (5)

⁎ Two were aged 45–59.

### 2.5 Ethical Issues

Written informed consent was obtained for all participants. Where people with CCD lacked capacity to give informed consent a personal consultee (relative) was appointed to provide advice on their wishes [17]. People with CCD could opt to participate in observations, semi-structured interviews or both. Ethical approval was gained from the Yorkshire & The Humber – Leeds Bradford Research Ethics Committee ref. 18/YH/0145.

## 3 Results

Interviews (*n* = 37) were conducted, with thirteen people with CCD, 22 relatives and nineteen staff members. Interview length varied between nine and 122 min due to varying communication abilities and preferences of participants. Observations and informal conversations were conducted with twelve people with dementia and the relatives who accompanied them, eight of these also participated in an interview. Most participants were recruited via NHS sites (see Table 1).

A total of 9 h of non-participant observations of clinical areas were conducted to explore routine patterns and use of the oncology departments, including waiting rooms, the radiotherapy department and outpatient clinics. Forty-six hours of participant observations were conducted.

The critical role of supportive networks was one of the major themes identified in the larger study. Within this theme four main themes, ‘reliance on family support’, ‘ability of family to support’, ‘the impact of providing support’ and ‘what if there is no support?’ and a number of sub-themes were developed. These are summarised in Table 2 and discussed below.

**Table 2:** Summary of main and subthemes.

**Main theme**	**Sub themes**
**Reliance on family support** – to access cancer treatment and care	**Practical support** – was required to attend appointments, manage symptoms and ‘fill the gaps’ that could occur as a result of memory loss
	**Emotional support** – including reassurance was provided during treatment and attendance at hospital appointments
	**Obligation and expectation to support** – families felt an obligation to provide the required support, sometimes even if they were a distant relative
**Ability of family to support** – families had varied abilities and resources to provide support needed	**Extent of family network** – some family networks were small with support falling to a small number of members who could feel alone
	**Physical ability to provide support** – some relatives, particularly spouses, may also have health problems or physical and/or cognitive frailty which limited their ability to provide practical support
	**Willingness to accept help** – some people with CCD were not welcoming of the support relatives wished and needed to provide
	**Knowledge of the person and their wishes** – in some cases families were unsure what the person with cancer or dementia would want with regard to treatment, when they were unable to express this for themselves
	**Harmony or conflict among family members** – while in some cases families came together to provide support, in others there was conflict around treatment and care decision-making
**The impact of providing support** – providing support had a range of usually negative impacts on families	**Guilt, stress and worry** – families often felt guilt, stress and worry, particularly when they had a central role to play in decision-making around cancer treatment and care and when facing extended periods of hospital attendance for treatments. Some families felt alone and unsupported at times.
**What if there is no family network?** – not everyone had a family network they could call upon to provide support.	**Who fills the gaps?** - When there was no family network it was unclear whose responsibility it was to fill the gaps.
	**Bouncing or assuming responsibility** – This often led to the person being ‘bounced around’ the system as different health and social care services argued about whose responsibility this was and who would pay for any required support

### 3.1 Reliance on Family Support

Relatives played crucial supportive roles in the provision of practical and emotional support for a person with CCD, which patients and oncology staff often relied upon. Many people with CCD, and their relatives, felt the person would be unable to attend oncology appointments unaccompanied. Relatives also regularly provided support with other practicalities (such as undressing and dressing) before and after treatment:

*PL0039: unbelievably hard, if had to make my own way there you know.*

*CL0040: I don't actually know how he'd get there because I don't really. Because he doesn't know where we are going.*

(Interview man with CCD PL0039 and daughter CL0040)

Dementia was felt to place more reliance on relatives for practical support than for people with cancer alone:

it's just another factor to put in, that … because of the dementia, there's more for me to do, to do with the prostate cancer, that it would be managed by C008 [man with prostate cancer and dementia] himself*.*

(Interview carer C009)

Relatives were often relied upon to the ‘fill the gaps’ created by the memory problems arising from dementia, including monitoring and reporting symptoms and side-effects and retaining and relaying information from oncology appointments to the person:

I can't see how you would ever be able to treat someone with dementia, if you didn't have, sort of, support from either an advocate, or a carer, or a family member. Because if these patients can't verbalise any problems, then it's dangerous giving people treatment*.*

(Interview lung clinic Clinical Nurse Specialist SB005)

CL0040: … obviously if I didn't go to appointments with him. He wouldn't know, what was going on really*.*

PL0039: well the doctors, go pretty fast don't they. They whiz you through it so I wouldn't remember it, when I come out I don't know what the doctors have said really*.*

(Interview man with CCD PL0039 and his daughter CL0040)

Families also provided emotional support and reassurance during treatment:

I: But you prefer it if he's [husband] there?

P:I feel safer with him*.*

(Interview woman with CCD PL002)

But just that familiar sound of somebody's voice. … I've done it a few times [have the family member in the radiotherapy room] … it worked really well for [patient with dementia] because she would keep still because he [husband] kept telling her to stay still. She obviously remembered who he was as opposed to us that she'd never met before*.*

(Interview Radiographer SL022)

Families discussed feeling an obligation to provide the required support:

Well you have to do don't you. You do. It's your family so you do it. You can't not

(Interview carer CL0040)

However, for more distant relatives this could entail an unexpected and perhaps unwelcome obligation:

And I think the cousin felt a little bit like, I suppose it's all down to her. That's a lot of responsibility for her to deal with, as a cousin. As a daughter or a son, it's sort of expected, isn't it? But as a cousin, it's different*.*

(Interview Lung Clinical Nurse Specialist SL003)

At times it seemed there was an expectation by oncology staff that relatives would be willing and able to deliver intimate care tasks; in this case, that a wife would give an enema to her husband prior to prostate radiotherapy:

‘I was just thinking, I don't think he'd be able to do an enema himself with having dementia’ says CL0036 [wife of PL0035 with prostate cancer and dementia]. The doctor replies to CL0036 ‘You'll be there’

(Field notes from observations of participants PL0035 and CL0036)

In other cases, staff assumed the person with dementia would be able to manage their own care needs at home, but in reality this was not always feasible, leaving relatives providing intensive input, sometimes with limited support:

I was saying to him. Right dad, you know what you are doing and he's going “erm. Erm. Erm.” Just couldn't do it [manage his catheter]. I was coming up [to his house to help] breakfast, dinner, tea. … sometimes with my dad if you keep going and going it does eventually get it but by the end of the 7 nights I was no further forward than in the beginning and I said to him this just isn't… it's never going to work

(Interview daughter carer CL0040)

### 3.2 Ability of Family to Support

Families varied in their ability to provide the types and levels of support needed. On a practical level this was dependent on the extent of the family network, their physical ability to provide support and whether or not the person with CCD was open to help:

… his [patient with CCD] wife was blind, so he was her carer and it turned out that he did have dementia, and he'd got in the car and set off, forgotten how to get here and got completely lost. She was shouting at him, calling him all names under the sun. He was upset. Obviously, he didn't want to accept that he needed help, because he was her carer*.*

(Interview Radiographer SL0025)

When support around treatment decision-making was required, the ability of families to act successfully in this role was dependent on their knowledge of the person and their wishes, alongside harmony or conflict among relatives:

You just sometimes think, I'm not sure that this patient would actually want all this doing. Then… if you get conflict in families as well*.*

(Interview urology Clinical Nurse Specialist SL007)

There's a lot to think about and I got really stressed with it, because I thought, everyone will want an input, because I've got family and I have to tell them and they might push to say, well she should have the operation, … But suddenly when you've got family, everyone has got an opinion, but they don't know the whole picture*.*

(Interview carer daughter CL0011)

Some relatives reported feeling alone with managing the support. However, others commented the support available within oncology services for patients and families was extensive and ongoing, in contrast to that experienced following a dementia diagnosis:

CB002: went for [dementia] tests at [name of hospital] but that was six months after we initially went to see Dr [name]. Then once we had the results of those tests back, nothing really happened from that point on*.*

CB002: we got her in to see this err, locum, [related to her cancer diagnosis] within two or three minutes he was like ‘right you're going down to [name of hospital] for an X-ray. …Next morning they rang us and we had to go to [name of hospital] to see the nurses down there and it all kicked off*.*

(Interview woman with CCD PB001 and husband CB002)

The complex needs and caregiving challenges associated with dementia made supporting someone with CCD additionally stressful.

And she was getting out of bed and forgetting she can't walk to the toilet and I was sleeping on the couch throughout the night and it just had to stop when I just passed out when they said it was stress

(Interview daughter CB0016)

Therefore, some family networks were better equipped than others to provide the necessary support. The majority of families appeared to access little external support for the person's cancer care needs, beyond that provided by General Practitioners as and when required and short-term support for specific aspects of healthcare need (e.g. District Nurses for dressings and wound care, incontinence nurses for catheter care and advice on incontinence products). No families discussed accessing specific cancer support beyond this, for example support provided by charities (except for the small number of participants recruited via a cancer support group). Support for dementia care needs was frequently described as absent, poor or as not meeting families' needs.

*She [dementia support worker] gave us some useful telephone numbers we may need for age concern and this sort of thing. So, yeah to say I*'*m disappointed I know its incurable and there*'*s not a lot they can do but just to be sort of sent off and told come back in a year and come back when she starts getting worse again you know. I found that hard to take.*

(Interview husband CL0024)

### 3.3 The Impact of Providing Support

Providing support to a person with CCD had a range of, usually negative, impacts on relatives. These included feelings of guilt, stress and worry; feelings not always shared by the person being cared for.:

I felt a kind of betrayal that I was betraying him by actually having to tell people his symptoms. Grassing him up kind of, do you know what I mean? That he couldn't tell people those things because if a Consultant or anybody asked him where his pain was or if pain had increased or anything, he always said to his knee or his colostomy bag. And I had to intervene*.*

(Interview wife CC002)

*I: So, do you feel like it's had an impact on both of you?*

*CB013: Yeah, it's stressful, isn't it?*

CB014: Yeah,.. I mean, now that we know what's going to happen and sort of, how long, and that they're going to monitor [rather than treat] her, it's a relief, isn't it? But before we said, what if they say she has to have it [treatment]? … How will she do that?

(Interview daughters CB013 and CB014)

Relatives reported at times feeling alone and unsupported:

we are now 6 months down the line from there and she just about getting back to where she was before she had this second tumour so. Erm, yeah. That's where we are now. I'm in full-time carer, there's nobody else that helps*.*

(Interview husband CL0024)

A small number of participants mentioned accessing external support such as community-based support groups, home care and district nursing. While for some these groups were extremely beneficial, for others they did not provide the combined support needed for people with CCD. For example, support groups attended were largely single disease specific, or were not able to accommodate the specific needs of people with dementia.

it's for what you call it. Young dementia hub. They don't call it a station, they call it a hub. Anyway, what they do. Everybody what goes there, everybody got some kind of dementia. One of my mates, … comes twice a week

(Interview man with CCD PL0035)

that's another [cancer specific] group that we tried once a month. But then I was so tired after looking after the children and I … couldn't face just going out again. … so I dropped him off on his own. It didn't work for him. … Being deaf, … speakers are … not used to projecting their voices. Sometimes [when they are] talking … he'd no way of getting it [understanding the discussion], and so after a couple of times going on his own he just said, ‘I don't think I want to go anymore’.

(Interview daughter CC009)

When home care or community-based healthcare serves were mentioned, this was commonly to discuss difficulties with accessing the care needed, sometimes because this was not able to be co-ordinated via the oncology department.

I had to fight a bit for the district nurse but I don't know that's not really the hospital is it. … I had to sort of say, yeah I do need somebody you know. And she [district nurse] said well we have to do it [provide catheter care] for so long and then it's not something we will do long term

(Interview wife CL0040)

Although services could make a significant difference when they were able to meet the unique needs associated with this comorbidity.

… when he actually went into the hospice, he just went for 4 weeks because at that point when I'd got to where I'd put it was unmanageable, they asked me if I would like him to go in to see if they could look at his pain level and see if they could manage it better. He loved going to the hospice, he loved the day centre, he loved everything about it

(Interview wife CC002)

Thus, external support, while accessed and valued by some participants, was often found to be difficult to access, not able to meet the needs of those with CCD, or not accessed at all by others.

### 3.4 What if there is no Support?

Not everyone with CCD had a family support network. Staff outlined the specific challenges this brought, including difficulties obtaining information and logistical difficulties:

Occasionally, if they're in a nursing home, they'll have an escort with them. If the escort would be a staff member, they don't send an escort. For a lot of the times from nursing homes, we find that escorts haven't travelled*.*

(Interview patient transport officer SL0021)

[Radiographer] spoke to me about a patient they treated last year with dementia. ‘We had a really bad case last year. He couldn't get an escort, his wife was housebound and patient transport was difficult. We asked them to bring him up to us in the department but we lost him a couple of times. It was really difficult.’

(Observation field notes PL0029 and CL0030)

For unaccompanied people it was unclear who could fill these gaps. Staff who might act in supportive roles often did not know the person well enough to provide the needed input, for example, into decision-making.

… when we use the IMCAS [Independent Mental Capacity Advocate Service], my experience has not always been good with them. I think it's good if they know the patient very well, and if they've been a carer and very involved. But it's very rare that you get that*.*

(Interview breast care nurse SB007)

When staff attempted to identify alternatives to family support it was very difficult to source:

There is no one to support this sort of thing [accompanying someone to hospital cancer treatment]. There is no, sort of, health related support workers. There was a health support worker that the [Local NHS Trust] agreed to put in place at one point… so we requested that they provided some support around escorting. But again, they didn't seem to see it was their role*.*

(Interview social worker SL0013)

Consequently, oncology staff often had to identify alternative solutions and find time to support unaccompanied people with CCD themselves, to avoid them missing out on cancer treatment.

## 4 Discussion

Few studies have examined the care and support needs of people with CCD, despite their complex medical and care needs and recognition that multi-morbidity in cancer care requires specific consideration [18]. While existing studies on CCD have focussed predominantly on the role of families in cancer decision-making, our study has demonstrated the vital role supportive networks play in enabling people with dementia to access and receive hospital-based cancer treatment and care. In line with literature from both cancer and dementia fields, support is provided by relatives, who give a range of practical and emotional help [[19], [20], [21]].

Families felt obliged to provide support for their relative, while oncology staff largely expected them to meet care needs that the person could not meet themselves. Caregiver obligation and willingness may impact caregiver coping, burden and health and has been explored in dementia literature [[22], [23], [24], [25]]. However, it remains relatively unexplored in cancer care [26]. There are recommendations that family carer capacity and readiness to undertake care tasks needs to form a central clinical priority in the integration of family carers into cancer healthcare systems [27]. Expectations clinical staff place on caregivers is under researched and appears to indicate an unexplored contributory factor for caregiver stress and burden.

Existing literature indicates that caregiving experiences in cancer are unique compared to those in other chronic conditions, due to rapid health deterioration often leading to intense care needs and the requirement for careful monitoring of symptoms. This has significant impacts on caregiver health and stress [27]. Our study suggests these needs are amplified when someone also has CCD. Relatives perceived CCD to have broader and greater impacts on them than cancer alone would, due to additional difficulties with memory, communication, behaviours, and daily activities, with acute, intense care needs associated with cancer potentially tipping the balance of coping. Thus, carers of people with CCD have specific additional needs to those managing each condition singularly and may be at greater risk of stress and harm. However, to date their needs have largely been unrecognised, although there is ongoing research in this area [28].

We encouraged participants to talk about their cancer care experiences/stories and what had been challenging or helpful. Whilst we did not specifically ask whether they were accessing community-based oncology support, meaning some people could have been accessing support they did not discuss, participants spoke extensively about their cancer care experiences and support needs, typically with little or no reference to community-based support. Our data suggests that where accessed single disease groups may offer some valuable support but may not be able to effectively address the complex needs associated with this comorbidity. It may be beneficial for oncology staff to better signpost carers to available services and support within their locality. Likewise, local providers of cancer support may need to consider how they can better accommodate the needs of those with dementia within their support groups and services. The challenges identified in accessing district nursing and escorting services for people with cancer and dementia, suggest there may also be limited provision that addresses some of the specific support needs that may arise for people with this comorbidity.

Our study identified that when people with CCD have few or no family supportive networks, providing care is particularly challenging, with staff relied upon to fill the gaps. Such individuals were at high risk of not being able to access cancer treatments if alternative support was not identified and may provide one explanation for the reported lower cancer treatment rates in people with dementia [5]. Existing research on the needs of people living alone with cancer does not consider individuals with extensive self-care needs and focuses on the balance between provision of support and maintenance of independence [[29], [30], [31], [32]]. The literature on living alone with dementia acknowledges the challenges individuals may face in caring for their own health and well-being [33] in accessing required services and support [34] and the difficulties professionals may face in meeting support needs considered to be outside of their role [35]. It highlights the need for more research to understand the care and support needs of this population [33,36]. Our study is the first to provide insight into the interaction between living alone, or with limited support networks, with CCD and indicates the additional needs and greater impact of this comorbidity on the individual and professionals supporting them than with single conditions alone.

Our study is one of the first to examine the cancer care and support needs of people with CCD and a range of cancer types, alongside that of their relatives and oncology staff, across more than one NHS Trust and using multiple data sources. Limitations of the study include a relatively small sample of largely white, British participants in one geographical area of the UK. Despite efforts to recruit a diverse sample, there was limited diversity among participants with CCD with regards to cancer types and treatments accessed (predominance of participants with lung cancer and experiences of outpatient-based treatments such as radiotherapy). Future research might benefit from capturing broader treatment experiences e.g. surgery which may require in-patient care. Likewise, our study did not specifically seek to ask about engagement with primary care and community-based support services or the interface of oncology with such services. Future research may benefit from seeking to include an understanding of these experiences, including the voices of professionals working in these services.

In summary, our study has offered significant new insights into the experiences and unique and complex needs, of people with CCD and the networks who support them. Oncology services need to assess and understand the supportive networks available to individual patients with CCD and relatives' willingness and ability to undertake supportive roles. The additional stress and personal impacts of caring for someone with CCD need greater consideration, including support for the family network as well as the patient. Greater clarity regarding support for people with CCD who have limited or no family support networks, and approaches for supporting them, should be a priority area for immediate consideration given the potential for cancer treatments to be inaccessible for these individuals.

## Declaration of Competing Interest

None.
